# Efficacy of Oxyclozanide and Closantel against Rumen Flukes (Paramphistomidae) in Naturally Infected Sheep

**DOI:** 10.3390/ani10111943

**Published:** 2020-10-22

**Authors:** David García-Dios, Pablo Díaz, Miguel Viña, Susana Remesar, Alberto Prieto, Gonzalo López-Lorenzo, Jose Manuel Díaz Cao, Rosario Panadero, Pablo Díez-Baños, Ceferino Manuel López

**Affiliations:** Investigación en Sanidad Animal: Galicia (Grupo INVESAGA), Department of Animal Pathology, Faculty of Veterinary Medicine (Universidade de Santiago de Compostela), 27002 Lugo, Spain; dgarciadios@gmail.com (D.G.-D.); mivivaz@gmail.com (M.V.); susana.remesar@usc.es (S.R.); alberto.prieto@usc.es (A.P.); gonzalolopezlorenzo@gmail.com (G.L.-L.); jmdchh@gmail.com (J.M.D.C.); rosario.panadero@usc.es (R.P.); pablo.diez@usc.es (P.D.-B.); c.lopez@usc.es (C.M.L.)

**Keywords:** sheep, paramphistomidae, oxyclozanide, closantel, efficacy

## Abstract

**Simple Summary:**

Paramphistomosis, caused by ruminal trematodes, is currently considered an emergent disease in Europe due to the progressive increase of prevalence and reports of acute cases in ruminants. Thus, finding effective control methods such as efficient drugs against paramphistomids is currently a priority. Although some drugs have been found effective for treating paramphistomid infections in cattle, data in sheep are currently limited. A single administration of oxyclozanide (15 mg/kg) or closantel (10 mg/kg) has been proven efficient in cattle, but it has not been tested in sheep; thus, the aim of the present study was to assess the effectiveness of these treatment protocols against paramphistomids in naturally infected sheep. The treatment with oxyclozanide was very efficient since it significantly reduced the paramphistomid egg output in more than 90% during 11 weeks after treatment. In contrast, closantel showed low efficacies throughout the study. The results of this study support the use of a single oral dose of oxyclozanide at 15 mg/kg for treating paramphistomid infections in sheep, whereas a single oral dose of closantel at 10 mg/kg is not effective.

**Abstract:**

Paramphistomosis is considered an emergent disease of ruminants in Europe. Some drugs have been found effective for treating paramphistomid infections in cattle, but data in sheep are currently limited. Thus, faecal samples from 25 adult sheep naturally infected with paramphistomids were collected weekly to test the efficacy of oxyclozanide and closantel. Three groups were performed: nine animals orally treated with a single dose of oxyclozanide (15 mg/kg bodyweight (BW) integrated the G-OXI group, whereas eight sheep orally treated with a single dose of closantel (10 mg/kg BW) were placed in a group called G-CLS. Eight untreated controls constituted the group G-CON. Oxyclozanide showed efficacies up to 90% until week 11 post-treatment, with a maximum efficacy of 98.3%, and significant differences were found between G-OXI and G-CON until the 26th week post-treatment. Closantel was insufficiently active (0–81%) throughout the study and differences compared to G-CON were never found. The present study reveals that oxyclozanide given at a single oral dose of 15 mg/kg BW is highly effective against adult rumen flukes in sheep. In addition, the use of a single oral dose of closantel at 10 mg/kg BW is not recommended for treating paramphistomid infections in sheep.

## 1. Introduction

Paramphistomids are worldwide distributed ruminal trematodes within the family Paramphistomidae [1]. *Calicophoron daubneyi* has been identified as the major paramphistomid in Europe, whereas *Paramphistomum leydeni* has also been occasionally found in sheep and wild ruminants from different European countries [2,3]. These parasites have an indirect life cycle with mud snails as intermediate hosts and both domestic and wild ruminants as definitive hosts [1,3,4,5,6]. These definitive hosts eliminate Paramphistomidae eggs in their faeces, contaminating the environment [1,7]. After hatching, miracidia infect a suitable intermediate host where it undergoes several stages of asexual reproduction (sporocyst, redia and cercaria) [1,7]. Finally, cercariae are released from the snail and encyst on the environment as metacercariae; ruminants get infected through ingestion of metacercariae with feed or water, and excysted juvenile stages considerably damage the duodenum mucosa [7]. In contrast, adult flukes, located in the rumen and reticulum, are relatively well-tolerated by the host [8,9] although there is evidence that they trigger an active immune response and mucosal morphological changes in the affected ruminal papillae [10,11]. Infected animals could show unspecific clinical signs as condition loss, anorexia, dehydration and watery diarrhoea, although severe and even fatal enteritis have also been reported in cases of heavy challenge [7,8,12].

The prevalence of paramphistomid infections in domestic ruminants from Europe has been steadily growing in recent years. Although these flukes were not considered important on the European continent, being mainly linked to tropical and subtropical areas, recent investigations have reported a noticeable increase of prevalences in different European countries [7]. This trend has been particularly evident in cattle from France [13,14], United Kingdom [5,15], Ireland [10], Italy [16] and Belgium [17] as well as in sheep, especially in Ireland [2,18] and Italy [16,19]. In Spain, several studies performed in cattle from northwestern regions confirmed the same trend as in other European areas [20,21,22,23]. 

The progressive increase of prevalence and reports of acute cases has led to the consideration of paramphistomosis as an emergent disease of ruminants in Europe [7,24,25]. It has been suggested that this emergence may be the consequence, among others, of changes in climate conditions, importation of infected livestock, availability of more accurate diagnostic techniques, continuous deworming with anthelmintics ineffective against paramphistomids and the good adaptation of the parasite to *Galba truncatula*, the major intermediate host in Europe [6,9,13,26]. In this new scenario, finding effective control methods is currently a priority including correct management of pastures and animals as well as the availability of efficient drugs against paramphistomids. Nevertheless, the most used anthelmintics in domestic ruminants such as macrocyclic lactones or benzimidazoles have showed limited or no effect in the treatment of these flukes [27,28,29]. Some drugs such as niclosamide, hexachlorophene and resorantel have showed a highly variable efficacy in cattle [28], being usually more active against adult flukes. The usefulness of closantel is controversial since high [27] and limited efficacy [17,28,29] has been reported in cattle. In contrast, most investigations reported oxyclozanide as highly efficient against both juvenile and adult paramphistomids in cattle and goats [28,30]. 

Works on the efficacy of drugs on paramphistomid infection in sheep are currently limited [31,32,33]. Only oxyclozanide, given orally at 20 mg/kg twice, three days apart, was proven effective in sheep [31]. Nevertheless, a single administration of a lower dosage of oxyclozanide (15 mg/kg) has been demonstrated highly efficient against rumen flukes in cattle, but it has not been tested in sheep. In addition, a single oral dosage of closantel at 10 mg/kg has been proven efficient in cattle [27] but no data on sheep are currently available. Thus, the aim of the present study was to assess the effectiveness of a single administration of oxyclozanide or closantel in naturally infected sheep. The results of the present study could be useful for implementing a cost-effective, less time-consuming and stressless treatment, which will be especially useful in flocks reared under extensive management.

## 2. Materials and Methods

### 2.1. Ethics Approval Statement

All faecal samples used in this study were collected with the permission of the farm owner. All experimental procedures fully complied with European and Spanish ethics regulations on the protection of animals used for scientific purposes (European Directive 2010/63/EU and Spanish Royal Decree 53/2013) and approved by the ethical committee of the University of Santiago de Compostela.

### 2.2. Experimental Design

The study was conducted on a small commercial mixed flock of sheep and goats dedicated to meat production located in Galicia (NW Spain). All animals were maintained in a semi-extensive husbandry system, where flocks went daily to pastures near the barn and kept indoors during the night. Based on pooled results gathered from previous six-monthly coprological analyses, this farm had been positive for paramphistomids in the last two parasitological controls. No effective treatments against paramphistomids had ever been administered to any animals; however, all adults were treated with ivermectin six months before this trial.

Firstly, all adult mixed-breed sheep (*n* = 36) were sampled and weighted, in order to obtain homogeneous groups. All 25 positive ewes (average shedding of 138.8 epg) were included in the study; these sheep were aged from 42 to 117 months, with live-weights ranging from 40 to 70 kg. Eleven of those positive animals also shed a small number of liver fluke eggs (average shedding of 12.7 epg). Using the sample() function in the R statistical package [34], positive animals were assigned to three groups showing a similar paramphistomid egg out-put:

− G-OXI: Nine sheep were orally treated with oxyclozanide (Rumenil^®^ 34 mg/mL, Karizoo, Spain) at a single dose of 15 mg/kg bodyweight.
− G-CLS: Eight animals received closantel (Endoex^®^ 5%, S.P. Veterinaria, Spain) at an oral single dose of 10 mg/kg bodyweight.
− G-CON: The remaining eight sheep were left untreated as controls.

Initial group sizes were slightly unbalanced but the number of animals per group fulfils the guidelines previously reported [35]. In addition, no statistical differences between groups considering the egg shedding intensity, age and weight were found using ANOVA test (*p* > 0.05). Each animal within those three groups was identified by a coloured necklace. The study started on November 2019 (Day 0) when the 25 animals included in the study were sampled and treated. Drugs were always administered by the same person with dosages based on the live-weight, rounded upwards to the nearest 10 kg. Faecal samplings were performed weekly during 11 weeks, with two additional samplings at 13 and 26 weeks after treatment, in order to cover the whole prepatent period of *C. daubneyi* [11,30], considered the major species of the Paramphistomidae family in Spain and in other European countries [2,3]. After treatment, animal management practices remained unchanged; the study animals were housed together with the rest of the flock and continued grazing in the same contaminated pastures. 

### 2.3. Faecal Samples and Analysis

Sampling and coprological analyses were always performed by the same investigators. Samples were collected directly from the rectum of the animals, kept at 4 °C until processing and analysed within 24 h. A quantitative sedimentation technique was used [36] and results were expressed in epg, with a sensitivity of 2.5 epg. Regarding the limited sensitivity of the sedimentation technique, animals were considered positive since the first sampling in which paramphistomid eggs were detected.

### 2.4. Assessment of Efficacy and Statistical Analysis

Geometric means were calculated for each group as recommended [35] in order to calculate the efficacy of each drug with the Henderson–Tilton formula [37]. This formula has been proven as the most appropriate for cases where the egg excretion of the control group fluctuates naturally during the experimental period [38]. The percentage of animals of each group shedding paramphistomid eggs was also assessed. To this end, an animal was considered positive since the first sampling it shed rumen fluke eggs.

Linear mixed effects models were carried out to evaluate whether the sampling effects of any of the two treatments were significantly different from the reference measure, i.e., control group in the day of treatment. The response variable was the log transform (ln (x + 1)) paramphistomid egg count; treatment and sampling week were fixed as effect variables; and the random variable was subject ID. Analysis was performed with lmer() function from lme4 package [39] in R statistical package [34]. *p*-values for mixed model were obtained with cftest() function from multcomp package [40]. Statistical differences were set at *p* ≤ 0.05.

## 3. Results

**Figure 1:**
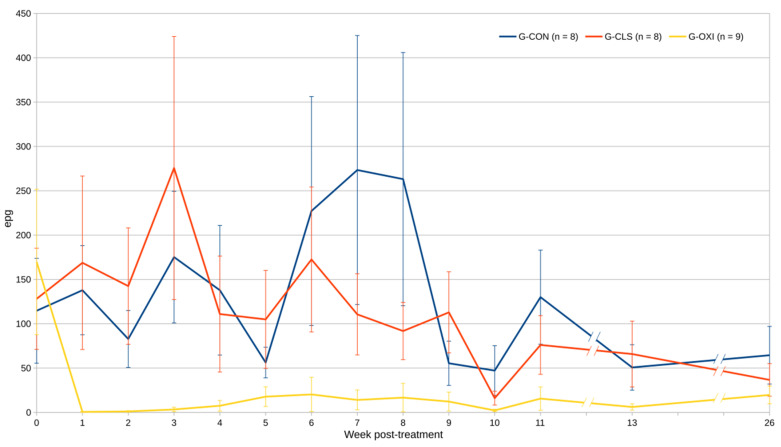
Arithmetic means and standard errors of paramphistomid egg shedding (eggs per gram, epg) in the three groups of sheep (G-CON, untreated controls; G-CLS, treated with closantel; G-OXI, treated with oxyclozanide) during the study period (26 weeks after treatment).

Lower and more stable mean egg counts were observed in G-OXI when compared to G-CLS and G-CON groups (Figure 1). 

**Figure 2:**
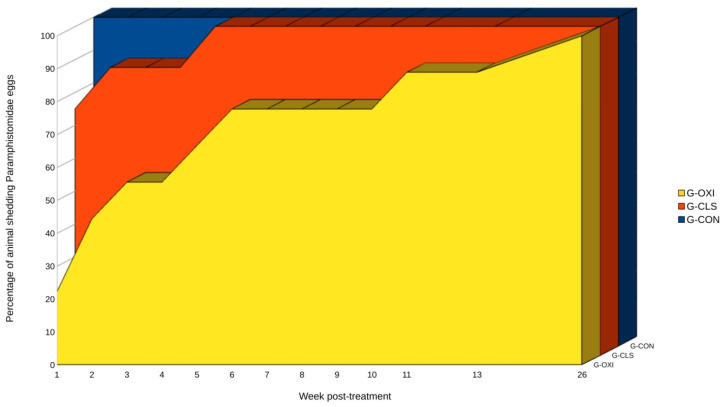
Percentage of animals shedding Paramphistomidae eggs detected in the three studied groups (G-OXI, G-CLS and G-CON) during study period (26 weeks after treatment). An animal was considered positive since the first sampling it shed paramphistomid eggs.

All G-CON sheep shed eggs during the study (Figure 2), but noticeable variations in the geometric mean egg counts were observed, with values ranging from 20.2 to 119.3 epg (Table 1). Animals from G-OXI excreted a mean number of eggs similar to G-CON before treatment (Figure 1 and Table 1). After the administration of oxyclozanide, only two sheep of G-OXI shed eggs in the first week post treatment (wpt) (Figure 2), leading to a significant reduction in the geometric mean values which remained below 3.7 epg during most of the study (Table 1). Sheep from this group became positive to paramphistomids progressively and all animals excreted eggs in the last sampling (Figure 2) where geometric mean of egg excretion intensity increased to 8.8 epg and no significant differences with G-CON were found. In contrast, six sheep from G-CLS eliminated paramphistomid eggs since the first wpt, and, in the fifth wpt all animals of this group were positive (Figure 2); geometric mean egg-shedding of G-CLS was always higher than those found in G-OXI and the linear mixed effects model showed no differences in egg counts between G-CLS and G-CON throughout the study (Table 1). 

The maximum efficacy of oxyclozanide (98.3%) was recorded in the first wpt, but effectiveness was above 90% until Week 11 (Table 1). The minimum efficacy was found in the last week of the study (74.9%). In contrast, after applying closantel, the reduction of egg shedding was under 80% throughout the study, except in the seventh wpt when it reached 80.4% (Table 1). Its minimum value (-33.8%) was reached in the ninth wpt.

## 4. Discussion

Geometric means of egg shedding in control group showed important variations throughout the study, ranging from 20 to 119 epg. These variations may be the consequence of natural fluctuations of paramphistomid egg shedding, including daily [4] and seasonal variations [21,41,42]. It is worth noting that a great increase on egg output was observed in all groups during seventh and eighth wpt, which corresponded to lambing season; although it has not been currently proven for trematode infections, these results may be related to a periparturient rise in faecal shedding of parasitic forms, as demonstrated for other parasites such as *Cryptosporidium* spp. [43,44] or gastrointestinal nematodes [45].

Our results reveal that a single dose of oxyclozanide at 15 mg/kg is very efficient against paramphistomid infections in sheep since a significant reduction in egg shedding was observed between treated and control animals and effectiveness remained above 90% during the first 11 weeks after treatment, complying with the international recommendations [35]. Since the prepatent period of *C. daubneyi* is considered to be 12–18 weeks [12,30], the absence of differences in egg output at the end of the study indicate that all animals have been re-infected. These results are consistent with those previously reported in domestic ruminants, showing efficiencies higher than 90% the first wpt when using a single dose ranging from 12.8 to 22.5 mg/kg BW in cows and goats [27,29,30]. Higher and longer efficacies have been reported when two doses are applied within a three-day interval [28,31] but, from an economic and management point of view, these results do not imply a significant improvement in efficacy when compared to those obtained using a single application. Our results suggest that further research using different dosages would allow optimising the effectiveness of the treatment.

Our data suggest that oxyclozanide eliminated all adults and juvenile flukes in a small percentage of animals, since they began to shed eggs after the prepatency period [11,30]. This finding suggests that this drug has some effectiveness against intestinal stages of paramphistomids, as previously reported [28,29]. However, the study design does not allow a reliable measuring of the effects of this drug against juvenile paramphistomids since the initial parasite burden in the duodenum and abomasum was not known. In contrast, more than 50% of sheep of G-OXI shed eggs in the fourth wpt or earlier, indicating that the drug had eliminated adults but not all juvenile paramphistomids. In addition, two animals were positive from the first wpt and efficacies of 100% were never recorded after a single treatment with oxyclozanide at 15 mg/kg, revealing that adult flukes were not completely eliminated in some animals. However, this study agrees with previous research reporting that oxyclozanide is an efficient and reliable anthelmintic for treating adult paramphistomids affecting domestic ruminants. In addition, our results show that G-OXI animals shed fewer eggs than G-CLS and G-CON, thus treating animals with oxyclozanide also reduces environment contamination with paramphistomid eggs and, consequently, infection in the intermediate host. Nevertheless, it is worth noting that an adequate control of rumen fluke infection in domestic ruminants should also involve suitable pasture management, including fencing in snail habitats, drenching or rotational grazing [46,47].

The efficacy of closantel in reducing paramphistomid egg output was low and irregular during the present study, never exceeding 81%; in addition, the percentage of positive animals was always over 70% after the treatment, and all sheep were positive in the fifth wpt. Thus, our data indicate that closantel was not efficient against both adult and juvenile rumen flukes in sheep when administered orally at 10 mg/kg. These results are in agreement with previous investigations reporting the absence of a significant reduction in the number of immature paramphistomids after treating cattle with a single dose of closantel at 7.5 mg/kg BW orally [28] and 10 mg/kg BW intraruminally [29]. In this regard, it has also been observed that a subcutaneous administration of closantel at 10 mg/kg was not efficient in reducing *C. daubneyi* egg shedding [17]. In contrast, the oral administration of this drug at single dose of 10 mg/kg BW was found effective against adult paramphistomids in adult cattle [27]. These discrepancies may be the consequence of using different administration routes [27]. Although using the same efficient deworming protocol previously reported in cattle [27], the low efficacy of closantel found in this study may be related to variations in the sensitivity of the detection techniques used, as well as to differences in drug pharmacokinetics or pharmacodynamics between sheep and cattle, as previously reported for other anthelmintics [48]. 

## 5. Conclusions

According to the present study, the situation of pharmacological control of paramphistomids in sheep is far from desirable. Oxyclozanide is the only anthelmintic showing a good performance against rumen flukes currently available in sheep; thus, strategic treatments together with complementary control measures for reducing pasture parasitic burden [47] are strongly recommended. Without other effective drugs available and rumen fluke prevalences increasing in Europe [7,49], finding new alternatives for the control of rumen flukes should be considered a priority.

## Figures and Tables

**Table 1:** Geometric mean egg counts in each group (G-CON, untreated controls; G-CLS, treated with closantel; G-OXI, treated with oxyclozanide) and efficacy of oxyclozanide and closantel throughout the study period (26 weeks after treatment). *p*-values for the Linear Mixed Effects Model (LMER) indicate if egg shedding in treated groups was significantly different from that in the G-CON before treatment.

	G-CON	G-OXI			G-CLOS		
Week Post-Treatment	Geometric Mean epg *	Geometric Mean epg *	Efficacy (%)	*p* (LMER)	Geometric Mean epg *	Efficacy (%)	*p* (LMER)
0	44.2	52.1			46.5		
1	64.7	1.3	98.3	<0.001	30.9	54.6	0.312
2	55.8	1.5	97.8	<0.001	34.8	40.7	0.5
3	72.9	1.8	97.9	<0.001	49.4	35.6	0.57
4	55.3	2.3	96.4	<0.001	16.5	71.6	0.104
5	43.2	3.7	92.8	<0.001	29.2	35.7	0.569
6	38.9	2.7	94.1	<0.001	28.2	30.9	0.715
7	119.3	2.6	98.1	<0.001	24.6	80.4	0.051
8	101.3	2.1	98.2	<0.001	43.6	59	0.304
9	25.1	2.6	91.3	0.001	35.3	Ineffective	0.638
10	20.2	1.9	92.1	<0.001	6.1	71.2	0.141
11	77.7	3.1	96.7	<0.001	26.7	67.2	0.19
13	20.3	3.2	86.7	0.011	14.2	33.1	0.96
26	29.7	8.8	74.9	0.141	11.5	63.2	0.641

* epg, eggs per gram of faeces.
